# Effect of kidney function on drug concentrations of first-line anti-tubercular treatment

**DOI:** 10.1007/s15010-026-02931-7

**Published:** 2026-08-31

**Authors:** Divya Datta, Indu Ramachandra Rao, Raghavendra Shetty, Shankar Prasad Nagaraju, Girish Thunga, Rahul Magazine, Shivashankara Kaniyoor Nagri, Nisha Abdul Khader, Pasumarthi Tejashree, Surulivelrajan Mallayasamy, Praveen Kumar Tirlangi, Dharshan Rangaswamy, Srinivas Vinayak Shenoy, Mohan V. Bhojaraja, Attur Ravindra Prabhu

**Affiliations:** 1 Department of Nephrology, Kasturba Medical College, Manipal Academy of Higher Education, Manipal, India; 2 Navitas Life Sciences Pvt. Ltd, Manipal, 576104 Karnataka India; 3 Department of Pharmacy Practice, Manipal College of Pharmaceutical Sciences, Manipal Academy of Higher Education, Manipal, India; 4 Department of Respiratory Medicine, Kasturba Medical College, Manipal Academy of Higher Education, Manipal, India; 5 Department of Medicine, Kasturba Medical College, Manipal Academy of Higher Education, Manipal, India; 6 Department of Infectious Disease, Kasturba Medical College, Manipal Academy of Higher Education, Manipal, India

**Keywords:** Chronic kidney disease, Tuberculosis, First-line anti-tubercular drugs, Pharmacokinetics, Drug concentration

## Abstract

**Background:**

Tuberculosis (TB) and chronic kidney disease (CKD) are interconnected global health concerns. CKD patients have a higher risk of developing TB. Decreased clearance of drugs in kidney disease enhances toxicity risk. Current guidance on medication doses is not based on therapeutic drug monitoring. We measured and compared the concentrations of first-line anti-TB drugs in CKD and those with normal kidney function.

**Methods:**

A prospective observational study was conducted from September 2020 to December 2023. Patients with microbiologically confirmed tuberculosis were included, and drug concentrations were determined by using Liquid Chromatography-Tandem Mass Spectrometer (LC-MS/MS) and compared between CKD and those with normal kidney function.

**Results:**

Of 151 participants, 51 had CKD. Higher trough concentrations were found in CKD for isoniazid [697.15 ng/mL (IQR 199.07–3321.68) vs. 187.58 ng/mL (IQR 95.56–323.65), *P* < 0.0001], rifampicin [141.53 ng/mL (IQR 40.86–730.66) vs. 33.24 ng/mL (IQR 22.49–86.20), *P* < 0.0001], and ethambutol [635.67 ng/mL (IQR 264.38–1594.12) vs. 230.92 ng/mL (IQR 161.95–417.22), *P* < 0.0001], while pyrazinamide level [1469.79 ng/mL (IQR 0.00–5160.53) vs. 4273.94 ng/mL (IQR 2151.29–8023.81), *P =* 0.002] was higher in those with normal kidney function. At 2-hours, a greater number of CKD patients had below the reference threshold for rifampicin [33/51 (64.7%) vs. 48/100 (48%), *P* = 0.059] and pyrazinamide [39/40 (97.5%) vs. 29/91 (31.8%), *P* < 0.0001].

**Conclusion:**

CKD affects anti-tubercular drug concentrations, highlighting the need for therapeutic drug monitoring.

**Clinical trial registration number:**

CTRI/2020/09/027951.

**Supplementary Information:**

The online version contains supplementary material available at https://doi.org/10.1007/s15010-026-02931-7.

## Background

Chronic kidney disease (CKD) is a global health concern, affecting 8.5 to 9.8% of the population, with increasing incidence, poor outcomes, and significant costs due to associated comorbidities, complications, and polypharmacy. By 2021, over 850 million people globally were estimated to have kidney disease, twice the number with diabetes mellitus and far exceeding the global prevalence of cancer and HIV/AIDS [1]. Whereas tuberculosis (TB) is a global health challenge with an incidence rate of 172 per lakh people in 2022 [2]. Current evidence substantiates that CKD is associated with a markedly increased susceptibility to active TB, as reflected by relative risk estimates ranging from 10 to 25.3 in comparison to the general population [3, 4]. The risk of TB increases by 5.1% for every 10 mL/min/1.73 m² decline in estimated glomerular filtration rate (eGFR), and a cohort from Taiwan further supports this link, showing a 27% higher incidence of CKD among individuals with TB [5, 6]. In patients with kidney failure, drug elimination is significantly impaired, leading to prolonged elevated plasma drug levels when standard therapeutic doses for patients with normal kidney function are used, which can increase the risk of toxicity.

Existing guidelines provide recommendations for dosing interval adjustment of pyrazinamide and ethambutol in patients with advanced renal impairment (eGFR < 30 mL/min/1.73 m²) and in those receiving dialysis, generally advising extended dosing intervals rather than dose reduction to preserve peak concentrations [7, 8]. No evidence-based dosing guidance exists for patients with mild-to-moderate CKD (stages 2–3), where eGFR-based thresholds for interval adjustment are not met, but pharmacokinetic alterations may already be present. The real and unaddressed gap therefore lies in individualised, therapeutic drug monitoring (TDM) guided dosing across the full spectrum of CKD severity a gap that cannot be filled by interval-adjustment rules alone.

The physiological disruptions associated with CKD, including reduced renal clearance, altered protein binding, changes in body composition, gastrointestinal dysmotility, and uraemia-related inhibition of hepatic enzyme activity, are well recognised to alter the absorption, distribution, metabolism, and excretion of multiple drugs [9]. For anti-tubercular agents, pharmacokinetic studies in patients with normal kidney function have already demonstrated substantial inter-individual variability in drug concentrations even at standard doses, with a significant proportion of patients failing to achieve established reference concentrations for rifampicin and isoniazid [10, 11]. However, data on these interindividual variations in clinical response, especially in patients with varying CKD stages, specifically stages two to five, are limited, indicating the need for further research in this area [12]. Therefore, we aimed to measure and compare concentrations of first-line anti-tubercular drugs in tuberculosis patients with CKD, specifically in stages two to five, and normal kidney function, to better understand drug concentration patterns in this population receiving guideline-based dosing.

## Methodology

### Study design and participant

After obtaining institutional ethical clearance (IEC-311/2020), a single-centre prospective observational study was conducted from 2020 to 2023. The study included consecutive microbiologically confirmed TB patients visiting the hospital. Patients were informed about the study procedures, and blood samples were collected after obtaining informed consent. Patients eligible for inclusion in the study were aged between 18 and 70 years and had been receiving first-line anti-tubercular medications for a minimum of two weeks prior to blood sample collection. Patients receiving renal replacement therapy, including haemodialysis or peritoneal dialysis, were excluded from the analysis. HIV-positive patients were excluded to avoid pharmacokinetic confounding from antiretroviral therapy interactions with rifampicin. Treatment was administered in adherence to the NTEP (National Tuberculosis Elimination Programme) guidelines, which carry WHO (World Health Organisation) endorsement for the diagnosis, management, and prevention of TB. The standard anti-tubercular regimen comprised two distinct phases: an intensive phase of two months followed by a continuation phase of four months. During the intensive phase, patients received once-daily isoniazid (300 mg maximum; 5 mg/kg/day), rifampicin (600 mg maximum; 10 mg/kg/day), ethambutol (1200 mg maximum; 20 mg/kg/day), and pyrazinamide (2000 mg maximum; 30 mg/kg/day). The subsequent continuation phase consisted of isoniazid (300 mg maximum; 5 mg/kg/day), rifampicin (600 mg maximum; 10 mg/kg/day), and ethambutol (1200 mg maximum; 20 mg/kg/day). Both single-drug formulations and fixed-dose combinations were employed based on clinical requirements [13]. For patients with CKD and an eGFR of < 30 mL/min/1.73 m², pyrazinamide and ethambutol were administered on alternate days and for these patients, blood samples were collected immediately before their scheduled dose, for trough concentration estimation. The primary endpoint was the comparison of trough and 2-hour post-dose plasma concentrations of first-line anti-tubercular drugs (isoniazid, rifampicin, pyrazinamide, and ethambutol) between patients with CKD and those with normal kidney function. Secondary endpoints included the proportion of patients with 2-hour concentrations below established reference thresholds and drug concentration variation across CKD stages. The analysis of treatment outcomes (treatment completion and mortality) was a pre-specified exploratory endpoint.

### Data collection

Demographic information, including age, gender, height, weight, medication history, type of medication, and other comorbidities, was recorded in a suitable case record form. CKD was defined as abnormalities of kidney structure or function, present for 3 months or more, with health implications. This includes either kidney damage or eGFR less than 60 mL/min/1.73 m². The eGFR was calculated using the “Chronic Kidney Disease Epidemiology Collaboration (CKD-EPI)” equation, and CKD definition and staging was as per the KDIGO 2012 guidelines [14]. Patients with acute kidney injury (AKI) or acute-on-chronic kidney disease were excluded from the study to ensure inclusion of only those with established, stable chronic kidney disease as per KDIGO 2012 criteria. Patients were interviewed using a validated questionnaire to assess their adherence to TB medication before sample collection. Only patients with confirmed adherence were included in the study. Participants were followed up till they completed their treatment. Treatment outcomes were classified in accordance with standard WHO/NTEP definitions as follows: Cure/ Treatment completed -bacteriological confirmation of negative sputum at the end of treatment and physician declared cured; Treatment failed- sputum positive at month 5 or later during treatment; Died- death from any cause during the treatment period; Lost to follow-up- treatment interrupted for two or more consecutive months. For the outcome analysis, a composite secondary outcome was defined: favourable outcome (cure or treatment completed) versus unfavourable outcome (treatment failed, died, or lost to follow-up).

### Sample collection

Patients were enrolled in the study at the time of microbiological confirmation of tuberculosis and initiation of first-line anti-tubercular therapy. Blood samples were collected during the second scheduled clinical visit, after a minimum of two weeks of therapy required before sampling to ensure steady-state drug concentrations had been achieved. The median time from treatment initiation to sample collection was 30 days (IQR: 18–47 days). Patients were requested to attend the hospital in a fasting state for pre-dose trough blood sample collection. Following trough sample collection, patients were permitted to have food and thereafter took their anti-tubercular medication at their usual time. A second venous blood sample was collected two hours after medication administration to measure the 2-hour post-dose concentration. For patients with eGFR < 30 mL/min/1.73 m² receiving pyrazinamide and ethambutol on alternate days, trough blood samples were collected on a dose day, immediately prior to the next scheduled dose administration; because dosing occurred every 48 h in this subgroup, the trough sample therefore reflects a 48-hour post-dose interval, compared with a 24-hour interval in patients receiving daily dosing. Blood was collected in EDTA-containing vacutainers wrapped in aluminium foil to protect the photosensitive anti-tubercular drugs. A trained phlebotomist performed the blood collection. Within one hour of collection, plasma was extracted through centrifugation at 3000 rpm for 10 min and then immediately stored at -70 °C until further analysis. A stabilising agent (Ascorbic acid) was added to the extracted plasma to avoid degradation of the drugs. Plasma drug concentrations were measured using trough and 2-hour post-dose blood samples. As full pharmacokinetic sampling was not performed, the 2-hour post-dose concentration was measured as a pragmatic clinical timepoint based on established TDM practice and previous studies [8, 15]. This two-point sampling design precludes calculation of full pharmacokinetic parameters; all inferences regarding drug concentrations should be interpreted as observational associations rather than pharmacokinetic conclusions. The threshold levels described in the literature were used to determine low 2-hour post-dose drug concentrations: 8000 ng/mL, 3000 ng/mL, 20,000 ng/mL, and 2000 ng/mL for rifampicin (RIF), isoniazid (INH), pyrazinamide (PZA), and ethambutol (ETB), respectively [11]. (Note: 1 µg/mL = 1000 ng/mL. Reference thresholds in µg/mL: Rifampicin ≥ 8 µg/mL; Isoniazid ≥ 3 µg/mL; Pyrazinamide ≥ 20 µg/mL; Ethambutol ≥ 2 µg/mL.)

### Drug concentration analysis

A validated LC-MS/MS technique, developed in conjunction with Navitas Life Sciences, Manipal, Karnataka, India, was utilised for the quantification of plasma concentrations of all four first-line ATT agents, including INH, RIF, PZA, and ETB. The method was validated for its accuracy, precision, lower quantification concentration (LQC), higher quantification concentration (HQC), extraction procedure, selectivity, stability and chromatographic condition. For chromatographic separation, isoniazid, pyrazinamide, and ethambutol were processed using the liquid-liquid extraction (LLE) method, while rifampicin was extracted using a solid-phase extraction (SPE) procedure. Following extraction, chromatographic separation and drug concentration measurement were performed using a Waters Acquity. Rifampicin drug concentration was measured using a Symmetry Shield Reverse Phase-C8 column (5 μm). The mobile phase comprised methanol, delivered at a flow rate of 0.600 mL/min, and each sample run time was 2 min. Isoniazid, pyrazinamide, and ethambutol concentrations were quantified utilising an Ascentis Express C18 column (5 cm × 4.6 mm, 2.7 μm), maintained at a column temperature of 40 °C with an injection volume of 5 µL. The mobile phase comprised 0.1% formic acid in methanol and 0.1% formic acid in water at a ratio of 70:30% (v/v), delivered at a flow rate of 0.300 mL/min, with a total run time of 2.5 min per sample. The Linearity range was 109.956 to 11995.200 ng/mL for Isoniazid, 101.589 to 25115.212 ng/mL for Rifampicin, 764.142 to 40218.000 ng/mL for Pyrazinamide, and 74.933 to 3996.400 ng/mL for ethambutol.

### Statistical analysis

All statistical evaluations were carried out utilising IBM SPSS Statistics (version 29; IBM Corp., Armonk, NY, USA). Categorical variables were depicted as frequencies and percentages, whereas continuous variables were conveyed as mean ± standard deviation or median with interquartile range, contingent upon their distributional characteristics. To assess differences in baseline characteristics between CKD patients and those with normal renal function, the independent t-test or Mann-Whitney U test was applied for continuous variables, while the chi-square test was utilised for categorical comparisons. Given the non-normal distribution of plasma drug concentrations, inter-group comparisons across CKD stages were conducted using the Kruskal–Wallis test, with subsequent post hoc pairwise analysis performed to identify differences between specific groups. Univariable and multivariable linear regression analyses were conducted to assess factors associated with drug concentrations, with log transformation applied to the dependent variables. Analysis was done keeping a log of ATT as dependent variables while other baseline characteristics like age, gender, BMI, Alcohol intake, smokers, serum albumin, diabetes mellitus, hypertension and CKD as independent variables. For sensitivity analysis, these regression models were repeated with eGFR entered as a continuous variable in place of CKD status, retaining the same remaining covariates as the corresponding primary model. To assess the magnitude of difference in delta drug concentrations (2-hour minus trough) between CKD and normal kidney function groups, the difference was calculated for each of the four first-line anti-tubercular drugs and expressed as median (IQR) in ng/mL. Additionally, univariate and multivariable logistic regression analyses were used to evaluate the relationship between ATT concentrations and clinical outcomes. ANCOVA was conducted on log-transformed trough (baseline) drug concentrations to assess differences between CKD and normal groups, adjusting for 2-hour concentrations as a covariate to account for shared population-level variance between sampling time points and to estimate the association between CKD status and trough drug concentrations independent of peak exposure. To assess effect sizes, partial eta squared (η²) was employed, which quantifies the proportion of the dependent variable’s variance explained by individual factors while controlling for other model variables. A p-value below 0.05 was considered statistically significant. Concentration–time profiles were analysed utilising R (version 4.4.3, R Foundation for Statistical Computing, Vienna, Austria). Graphical representation of the data was performed using the ggplot2 package to visualise drug concentration profiles over time. No adjustment for multiple comparisons was applied. The primary endpoint was the comparison of trough and 2-hour post-dose drug concentrations between CKD and normal kidney function groups. All other analyses, including stage-specific comparisons, regression analyses, and outcome analyses, are secondary or exploratory, and their findings should be interpreted as hypothesis-generating rather than confirmatory, given the risk of type I error from multiple testing.

## Results

**Fig. 1:**
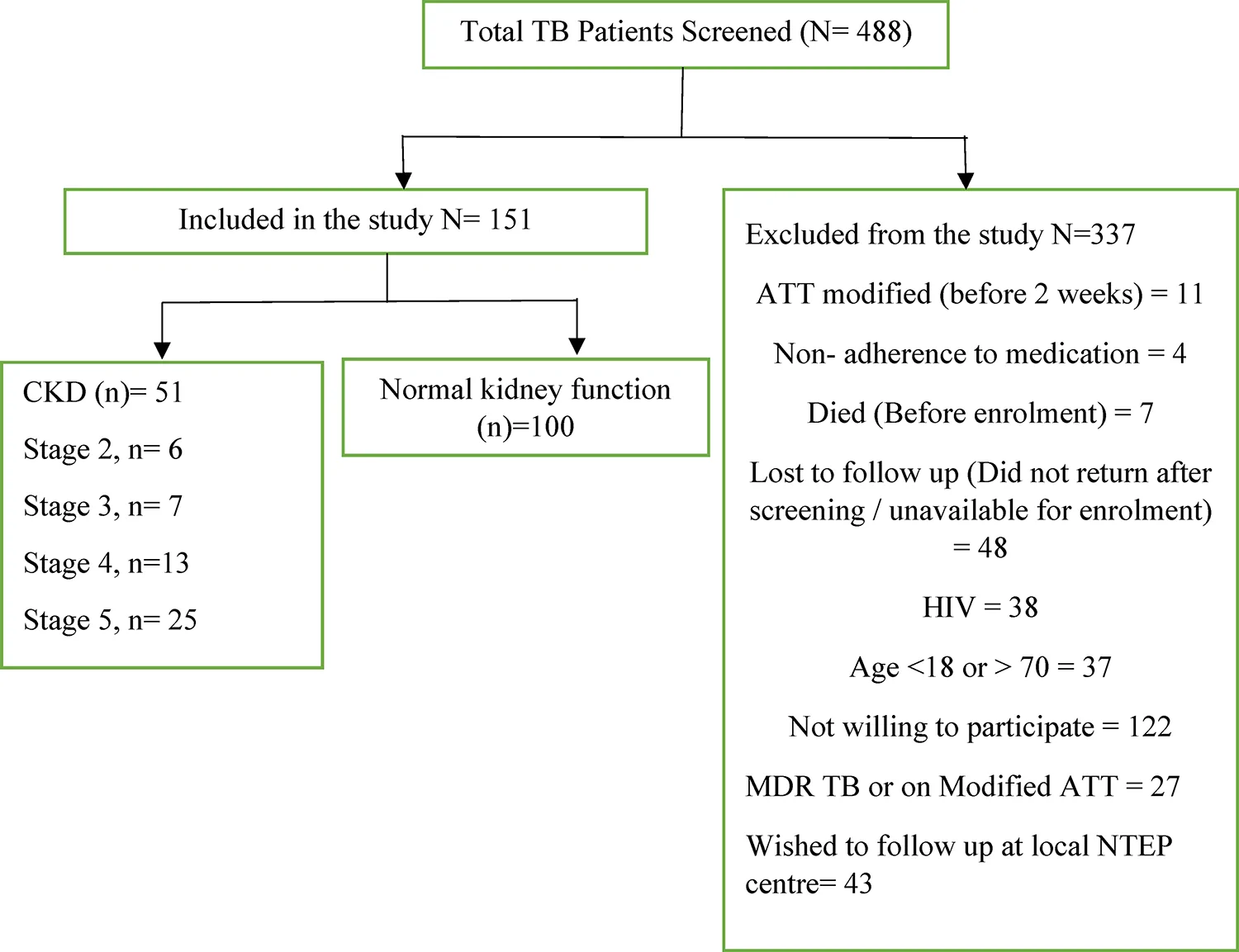
Flow diagram of the study participants

Of 488 patients screened, 151 were included. Of these, 51 had CKD, with 6 (4%), 7 (4.6%), 13 (8.6%) and 25 (16.6%) in stages 2,3,4 and 5 respectively (Fig. 1). Males [41 (80.3%) vs. 62 (62.0%), *P =* 0.027], diabetes mellitus [25 (49.0%) vs. 28 (28.0%), *P =* 0.012], hypertension [39 (76.4%) vs. 15 (15.0%), *P* < 0.0001] were more common in CKD compared to normal kidney function. The mean age was also higher in CKD [52.29 ± 14.48 vs. 44.23 ± 14.77, *P =* 0.002] (Table 1).

This figure illustrates the selection process and distribution of participants included in the study, showing the number of subjects screened, excluded, and analysed.

**Table 1:** Demographic details

Characteristics		CKD (*n* = 51)	Normal kidney function (*n* = 100)	*P* value
Male Gender, n (%)		41 (80.3%)	62 (62.0%)	**0.027**
Age (mean ± SD)		52.29 ± 14.48	44.23 ± 14.77	**0.002**
BMI (mean ± SD)		17.95 ± 9.01	18.86 ± 7.19	0.609
Diabetes Mellitus, n (%)		25 (49.0%)	28 (28.0%)	**0.012**
Hypertension, n (%)		39 (76.4%)	15 (15.0%)	**< 0.0001**
Alcohol intake, n (%)		7 (13.7%)	25 (25.0%)	0.141
Smoker, n (%)		5 (9.8%)	24 (24.0%)	**0.048**
Site of TB, n (%)	PTB	19 (37.2%)	52 (52.0%)	0.226
	EPTB	29 (56.8%)	44 (44.0%)	
	Disseminated	3 (5.8%)	4 (4.0%)	

*BMI: Body mass index; TB: Tuberculosis; PTB: Pulmonary Tuberculosis; EPTB: Extra pulmonary tuberculosis

### Drug concentration of first-line ATT

#### Isoniazid

Trough concentrations of isoniazid were significantly higher in CKD patients compared to those with normal kidney function [697.15 ng/mL (IQR: 199.07–3321.68) vs. 187.58 ng/mL (IQR: 95.56–323.65), *P* < 0.0001]. At 2-hours post-dose, isoniazid concentrations were not significantly different from CKD [4712.06 ng/mL (IQR: 1905.66–9271.04) vs. 3435.60 ng/mL (IQR: 2280.25–5456.88), *P* = 0.069] (Table 2). No significant difference was observed for the median delta (2-hour minus trough) concentration of isoniazid between the two groups (*P* = 0.998) (Supplementary Table-S1).

At trough level, significant differences in isoniazid concentrations were observed across CKD stages (*P* = 0.001); however, at 2-hours, isoniazid did not show statistically significant variation across CKD stages (*P* = 0.123). On post hoc pairwise comparisons, trough isoniazid concentrations were significantly higher in CKD stage 4 [1050 ng/mL (IQR: 357–4226) vs. 201 ng/mL (IQR: 105–330), *P* = 0.019] and stage 5 [2306 ng/mL (IQR: 675–4376) vs. 201 ng/mL (IQR: 105–330), *P* = 0.024] compared to normal kidney function. No significant differences in 2-hour isoniazid concentrations were observed across CKD stages (Supplementary Table-S2 & S3).

**Fig. 2:**
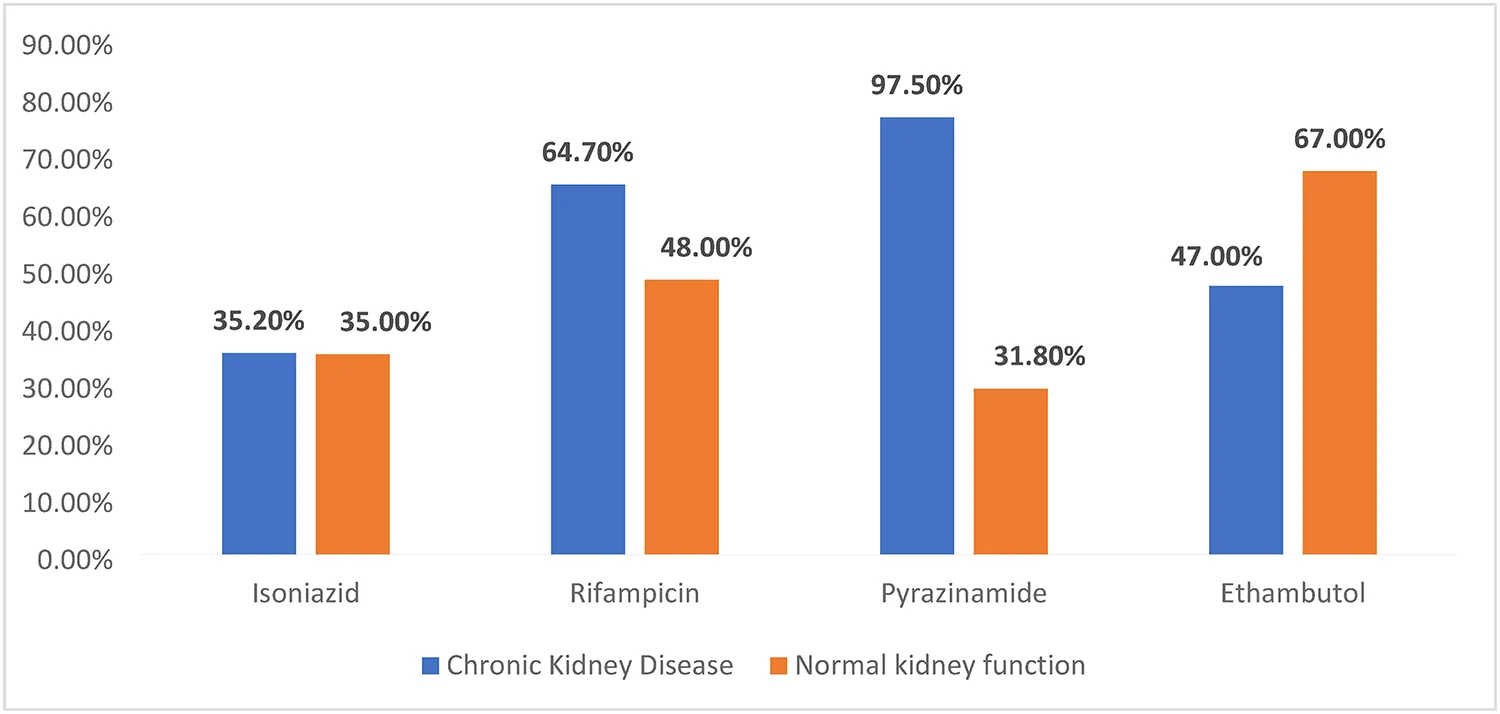
Prevalence of anti-tuberculosis (ATT) drug concentrations below the established reference threshold after a 2-hour dosage. Reference concentration ranges were adopted from previously published data by Trentalange et al., including Rifampicin (RIF)− 8000 ng/mL; Isoniazid (INH)− 3000 ng/mL; Pyrazinamide (PZA) − 20,000 ng/mL; Ethambutol (ETB)−2000 ng/mL [11]. 1 µg/mL = 1000 ng/mL. Reference thresholds in µg/mL: Rifampicin ≥8 µg/mL; Isoniazid ≥3 µg/mL; Pyrazinamide ≥20 µg/mL; Ethambutol ≥2 µg/mL [11]. 1 µg/mL = 1000 ng/mL. Reference thresholds in µg/mL: Rifampicin ≥8 µg/mL; Isoniazid ≥3 µg/mL; Pyrazinamide ≥20µg/mL; Ethambutol ≥2 µg/mL

The proportion of patients with 2-hour isoniazid concentrations below the reference threshold was similar between the CKD and normal kidney function groups [18/51 (35.2%) vs. 35/100 (35.0%), *P* = 1.000] (Fig. 2).

#### Rifampicin

Trough concentrations of rifampicin were significantly higher in CKD patients compared to those with normal kidney function [141.53 ng/mL (IQR: 40.86–730.66) vs. 33.24 ng/mL (IQR: 22.49–86.20), *P* < 0.0001]. In contrast, 2-hour post-dose rifampicin concentrations were significantly higher in the normal kidney function group [4097.97 ng/mL (IQR: 968.12–12851.87) vs. 8114.65 ng/mL (IQR: 3178.24–13602.36), *P* = 0.033] (Table 2). CKD patients exhibited a significantly lower increase in rifampicin levels (delta concentration: 2-hour minus trough) compared to those with normal kidney function (*P* = 0.009) (Supplementary Table-S1).

At trough level, significant differences in rifampicin concentrations were observed across CKD stages (*P* < 0.0001); at 2-hours, significant differences were also observed across CKD stages (*P* = 0.021). On post hoc pairwise comparisons, significantly higher trough concentrations were observed only in CKD stage 5 compared to normal kidney function [302 ng/mL (IQR: 89.9–795) vs. 36.2 ng/mL (IQR: 24.2–88.9), *P* < 0.0001]. At 2-hours, rifampicin concentrations were significantly lower in stage 5 compared to normal kidney function [3334 ng/mL (IQR: 988–7661) vs. 8115 ng/mL (IQR: 3191–13521), *P* = 0.039] (Supplementary Table-S2 & S3).

A higher proportion of CKD patients had 2-hour rifampicin concentrations below the reference threshold compared to those with normal kidney function [33/51 (64.7%) vs. 48/100 (48.0%), *P* = 0.059], though this difference did not reach conventional statistical significance (Fig. 2).

#### Pyrazinamide

Pyrazinamide concentrations were not available for 20 participants (11 with CKD, 9 with normal renal function) owing to non-intake of the drug at the time of sampling. All pyrazinamide analyses were therefore conducted using available data from 40 CKD patients and 91 patients with normal kidney function. Trough concentrations of pyrazinamide were significantly higher in the normal kidney function group [1469.79 ng/mL (IQR: 0.00–5160.53) vs. 4273.94 ng/mL (IQR: 2151.29–8023.81), *P* = 0.002]. Similarly, 2-hour post-dose pyrazinamide concentrations were significantly higher in the normal kidney function group [7685.76 ng/mL (IQR: 2251.79–41644.57) vs. 32216.62 ng/mL (IQR: 23267.01–42096.23), *P* = 0.001] (Table 2). CKD patients exhibited a significantly lower increase in pyrazinamide levels (delta concentration: 2-hour minus trough) compared to those with normal kidney function (*P* = 0.002) (Supplementary Table-S1).

At trough level, significant differences in pyrazinamide concentrations were observed across CKD stages (*P* < 0.0001); at 2-hours, significant differences were also observed across CKD stages (*P* < 0.0001). On post hoc pairwise comparisons, trough concentrations were significantly lower in stage 4 [3143 ng/mL (IQR: 2510–3414) vs. 4805 ng/mL (IQR: 3016–8138), *P* = 0.002] and significantly higher in stage 5 [5118 ng/mL (IQR: 2620–7233) vs. 4805 ng/mL (IQR: 3016–8138), *P* = 0.002] compared to normal kidney function. At 2-hours, pyrazinamide concentrations were significantly lower in stage 4 [3196 ng/mL (IQR: 1633–26587) vs. 32006 ng/mL (IQR: 23164–41233), *P* = 0.031] and stage 5 [7488 ng/mL (IQR: 1745–41569) vs. 32006 ng/mL (IQR: 23164–41233), *P* = 0.001] compared to normal kidney function (Supplementary Table-S2 & S3).

A significantly higher proportion of CKD patients had 2-hour pyrazinamide concentrations below the reference threshold compared to those with normal kidney function [39/40 (97.5%) vs. 29/91 (31.8%), *P* < 0.0001] (Fig. 2).

#### Ethambutol

Trough concentrations of ethambutol were significantly higher in CKD patients compared to those with normal kidney function [635.67 ng/mL (IQR: 264.38–1594.12) vs. 230.92 ng/mL (IQR: 161.95–417.22), *P* < 0.0001]. At 2-hours post-dose, ethambutol concentrations also remained significantly higher in the CKD group [2023.60 ng/mL (IQR: 1157.87–4489.23) vs. 1589.37 ng/mL (IQR: 838.42–2441.28), *P* = 0.015] (Table 2). No significant difference was observed for the median delta (2-hour minus trough) concentration of ethambutol between the two groups (*P* = 0.832) (Supplementary Table-S1).

At trough level, significant differences in ethambutol concentrations were observed across CKD stages (*P* = 0.001); at 2-hours, significant differences were also observed across CKD stages (*P* = 0.010). On post hoc pairwise comparisons, trough concentrations were significantly higher in CKD stage 3 [804 ng/mL (IQR: 624–1087) vs. 231 ng/mL (IQR: 163–415), *P* = 0.009] and stage 5 [1577 ng/mL (IQR: 400–2343) vs. 231 ng/mL (IQR: 163–415), *P* = 0.021] compared to normal kidney function. At 2-hours, significantly higher ethambutol concentrations were observed only in stage 5 compared to normal kidney function [3012 ng/mL (IQR: 1537–5234) vs. 1583 ng/mL (IQR: 852–2359), *P* = 0.002] (Supplementary Table-S2 & S3).

The proportion of patients with 2-hour ethambutol concentrations below the reference threshold was significantly higher in the normal kidney function group compared to CKD patients [67/100 (67.0%) vs. 24/51 (47.0%), *P* = 0.022] (Fig. 2).

**Table 2:** Comparison of first-line anti-tubercular drug concentration in CKD and normal kidney function

Drug, Median (IQR), ng/mL	CKD (*n* = 51)	Normal kidney function (*n* = 100)	*P* value
Isoniazid trough	697.15 (199.07–3321.68)	187.58 (95.56–323.65)	**< 0.0001**
Isoniazid 2-Hour	4712.06 (1905.66–9271.04)	3435.60 (2280.25–5456.88)	0.069
Rifampicin trough	141.53 (40.86–730.66)	33.24 (22.49–86.20)	**< 0.0001**
Rifampicin 2-Hour	4097.97 (968.12–12851.87)	8114.65 (3178.24–13602.36)	**0.033**
Pyrazinamide trough	*N* = 40 1469.79 (0.00–5160.53)	*N* = 91 4273.94 (2151.29–8023.81)	**0.002**
Pyrazinamide 2-Hour	*N* = 40 7685.76 (2251.79–41644.57)	*N* = 91 32216.62 (23267.01–42096.23)	**0.001**
Ethambutol trough	635.67 (264.38–1594.12)	230.92 (161.95–417.22)	**< 0.0001**
Ethambutol 2-Hour	2023.60 (1157.87–4489.23)	1589.37 (838.42–2441.28)	**0.015**

*CKD; Chronic kidney disease, ng/mL; Nanogram/millilitre; IQR; Interquartile range

Pyrazinamide levels were unavailable for 20 participants (11 with CKD, 9 with normal renal function) owing to non−intake of the drug at the time of sampling

1 µg/mL = 1000 ng/mL. Reference thresholds in µg/mL: Rifampicin ≥8 µg/mL; Isoniazid ≥3 µg/mL; Pyrazinamide ≥20 µg/mL; Ethambutol ≥2 µg/mL

The figure depicts the proportion of participants with plasma concentrations of ATT drugs below the established reference threshold, measured 2-hours post-dose.

### Association between CKD status and drug concentrations

The 2-hour concentration showed significant association with trough for isoniazid (*F* = 41.51; *P* < 0.001; partial η² = 0.237), rifampicin (*F* = 6.27; *P =* 0.013; partial η² = 0.043), pyrazinamide (*F* = 46.32; *P* < 0.001; partial η² = 0.325), and ethambutol (*F* = 94.19; *P* < 0.001; partial η² = 0.400). CKD status also remained a significant predictor of trough concentrations for isoniazid (*F* = 42.45; *P* < 0.001; partial η² = 0.241), rifampicin (*F* = 29.47; *P* < 0.001; partial η² = 0.174), and ethambutol (*F* = 48.61; *P* < 0.001; partial η² = 0.256), but not for pyrazinamide (*F* = 2.35; *P =* 0.128; partial η² = 0.024). The overall models explained ~ 45% of the variance for isoniazid (adjusted R² = 0.450), 17% for rifampicin (adjusted R² = 0.170), 32% for pyrazinamide (adjusted R² = 0.316), and 53% for ethambutol (adjusted R² = 0.534) (Table 3).

**Table 3:** Baseline Drug Concentrations Adjusted for 2-Hour Levels

Drug	Effect (Source)	F	*P*-value	Partial η²
Isoniazid	(2-Hour)	41.51	< 0.001	0.237
	CKD	42.45	< 0.001	0.241
Rifampicin	(2-Hour)	6.27	0.013	0.043
	CKD	29.47	< 0.001	0.174
Pyrazinamide	(2-Hour)	46.32	< 0.001	0.325
	CKD	2.35	0.128	0.024
Ethambutol	(2-Hour)	94.19	< 0.001	0.400
	CKD	48.61	< 0.001	0.256

1 µg/mL = 1000 ng/mL. Reference thresholds in µg/mL: Rifampicin ≥8 µg/mL; Isoniazid ≥3 µg/mL; Pyrazinamide ≥20 µg/mL; Ethambutol ≥2 µg/mL

### Factors associated with trough drug concentrations

In the case of multiple linear regression analysis of the trough levels of drugs, isoniazid was significantly high in CKD (β: 0.925, 95% CI: 0.326 to 1.524; *P =* 0.003) and hypertension (β: 0.663, 95% CI: 0.084 to 1.242; *P =* 0.025), while higher serum albumin level was related with significantly low concentrations (β: −0.413, 95% CI: −0.734 to − 0.091; *P =* 0.012). Rifampicin trough levels had significant negative correlation with serum albumin (β: −0.671, 95% CI: −1.045 to − 0.297; *P =* 0.001). Trough pyrazinamide levels were significantly low in the presence of diabetes mellitus (β: −0.642, 95% CI: −1.027 to − 0.257; *P =* 0.001). For ethambutol, trough levels were increased in those with CKD (β: 0.833, 95% CI: 0.420 to 1.246; *P* < 0.0001), while the trough levels were lower in those with diabetes mellitus (β: -0.659, 95% CI: -0.992 to -0.326; *P* < 0.0001) (Table 4).

In the sensitivity analysis using eGFR as a continuous variable, higher eGFR was significantly associated with lower trough concentrations of isoniazid (adjusted β = −0.012, 95% CI: −0.019 to − 0.005; *P* = 0.001), rifampicin (adjusted β = −0.009, 95% CI: −0.017 to − 0.002; *P* = 0.014), and ethambutol (adjusted β = −0.009, 95% CI: −0.013 to − 0.004; *P* < 0.001). No significant association was observed between eGFR and trough pyrazinamide concentration (adjusted β = −0.0001, 95% CI: −0.005 to 0.005; *P* = 0.997) (Supplementary Table-S4).

**Table 4:** Factors Affecting Trough Concentrations of Anti-Tubercular Drugs: Results from Univariable and Multivariable Regression Analysis

	Isoniazid		Rifampicin		Pyrazinamide		Ethambutol	
Covariates	β coefficient (95%CI)	*P* value	β coefficient (95%CI)	*P* value	β coefficient (95%CI)	*P* value	β coefficient (95%CI)	*P* value
Unadjusted Model								
Age	0.019 (0.003 to 0.034)	0.022	0.017 (0.0001 to 0.034)	0.049	0.002 (-0.010 to 0.015)	0.709	0.012 (0.0001 to 0.023)	0.042
Male- gender	-0.540 (-1.050 to -0.030)	0.038	-0.561 (-1.112 to -0.011)	0.046	-0.234 (-0.638 to 0.171)	0.254	-0.343 (-0.701 to 0.016)	0.061
BMI	0.002 (-0.037 to 0.040)	0.933	0.012 (-0.029 to 0.053)	0.563	0.003 (-0.030 to 0.037)	0.839	0.0001 (-0.026 to 0.026)	0.988
Alcohol intake	-0.246 (-0.553 to 0.062)	0.117	-0.044 (-0.380 to 0.291)	0.795	0.169 (-0.066 to 0.404)	0.157	0.038 (-0.182 to 0.257)	0.736
Smoker	-0.341 (-0.656 to -0.025)	0.034	0.027 (-0.319 to 0.373)	0.877	0.123 (-0.108 to 0.354)	0.294	-0.055 (-0.281 to 0.171)	0.633
Diabetes Mellitus	0.195 (-0.307 to 0.698)	0.444	0.442 (-0.092 to 0.976)	0.104	-0.610 (-0.998 to -0.223)	0.002	-0.279 (-0.637 to 0.078)	0.125
Hypertension	1.371 (0.927 to 1.815)	< 0.0001	0.946 (0.431 to 1.462)	< 0.0001	-0.006 (-0.424 to 0.412)	0.978	0.762 (0.431 to 1.092)	< 0.0001
CKD	1.636 (1.199 to 2.073)	< 0.0001	1.252 (0.747 to 1.757)	< 0.0001	-0.204 (-0.696 to 0.287)	0.411	1.063 (0.743 to 1.383)	< 0.0001
Albumin	-0.670 (-0.990 to -0.349)	< 0.0001	-0.810 (-1.147 to -0.474)	< 0.0001	-0.129 (-0.424 to 0.165)	0.386	-0.320 (-0.553 to -0.087)	0.007
Adjusted Model								
Age	-0.004 (-0.019 to 0.012)	0.633	-0.007 (-0.025 to 0.011)	0.463	-	-	0.004 (-0.007 to 0.016)	0.451
Male- gender	-0.219 (-0.703 to 0.266)	0.373	-0.311 (-0.847 to 0.225)	0.253	-	-	0.188 (-0.514 to 0.138)	0.256
BMI	-	-	-	-	-	-	-	-
Alcohol intake	-0.068 (-0.441 to 0.306)	0.721	-	-	0.203 (-0.022 to 0.428)	0.076	-	-
Smoker	-0.153 (-0.543 to 0.236)	0.437	-	-	-	-	-	-
Diabetes Mellitus	-	-	-0.058 (-0.596 to 0.480)	0.832	-0.642 (-1.027 to -0.257)	0.001	-0.659 (-0.992 to -0.326)	< 0.0001
Hypertension	0.663 (0.084 to 1.242)	0.025	0.406 (-0.263 to 1.076)	0.232	-	-	0.292 (-0.132 to 0.717)	0.176
CKD	0.925 (0.326 to 1.524)	0.003	0.649 (-0.014 to 1.311)	0.055	-	-	0.833 (0.420 to 1.246)	< 0.0001
Albumin	-0.413 (-0.734 to -0.091)	0.012	-0.671 (-1.045 to -0.297)	0.001	-	-	-0.195 (-0.423 to 0.033)	0.093

### Factors associated with 2-hour drug concentrations

In multivariable analysis of 2-hour drug concentrations, CKD was independently associated with significantly lower pyrazinamide concentrations (β: −1.509, 95% CI: −2.055 to − 0.963; *P* < 0.0001). Diabetes mellitus was independently associated with lower 2-hour concentrations of isoniazid (β: −0.320, 95% CI: −0.598 to − 0.041; *P* = 0.025) and ethambutol (β: −0.492, 95% CI: −0.782 to − 0.202; *P* = 0.001). Male gender was independently associated with higher 2-hour rifampicin concentrations (β: 0.591, 95% CI: 0.020 to 1.163; *P* = 0.043), and older age was independently associated with higher 2-hour isoniazid concentrations (β: 0.304, 95% CI: 0.019 to 0.588; *P* = 0.036) (Table 5).

In the corresponding sensitivity analysis for 2-hour concentrations, higher eGFR was significantly associated with higher 2-hour concentrations of pyrazinamide (adjusted β = 0.018, 95% CI: 0.012 to 0.024; *P* < 0.0001) and lower 2-hour concentrations of ethambutol (adjusted β = −0.004, 95% CI: −0.007 to 0.000; *P* = 0.037). No significant association was observed between eGFR and 2-hour concentrations of isoniazid (adjusted β = 0.001, 95% CI: −0.003 to 0.005; *P* = 0.711) or rifampicin (adjusted β = 0.006, 95% CI: −0.001 to 0.012; *P* = 0.087) (Supplementary Table-S5).

**Table 5:** Factors affecting two-hour concentrations of anti-tubercular drugs: Results from Univariable and Multivariable Regression Analysis

	Isoniazid		Rifampicin		Pyrazinamide		Ethambutol	
Unadjusted Model								
Covariates	β coefficient (95%CI)	*P* value	β coefficient (95%CI)	*P* value	β coefficient (95%CI)	*P* value	β coefficient (95%CI)	*P* value
Age	0.0001 (-0.009 to 0.009)	0.969	-0.008 (-0.025 to 0.009)	0.346	-0.013 (-0.027 to 0.001)	0.071	-0.002 (-0.011 to 0.008)	0.715
Male- gender	0.250 (-0.029 to 0.528)	0.078	0.748 (0.203 to 1.293)	0.008	0.300 (-0.169 to 0.770)	0.208	0.159 (-0.139 to 0.456)	0.294
BMI	-0.008 (-0.028 to 0.013)	0.457	-0.011 (-0.053 to 0.031)	0.601	0.003 (-0.038 to 0.044)	0.897	0.007 (-0.014 to 0.028)	0.501
Alcohol intake	-0.049 (-0.219 to 0.121)	0.568	-0.015 (-0.351 to 0.321)	0.929	0.130 (-0.151 to 0.411)	0.363	0.022 (-0.159 to 0.202)	0.814
Smoker	-0.069 (-0.244 to 0.106)	0.439	-0.027 (-0.373 to 0.320)	0.880	0.216 (-0.064 to 0.495)	0.129	-0.022 (-0.208 to 0.165)	0.819
Diabetes Mellitus	-0.212 (-0.484 to 0.060)	0.125	-0.306 (-0.846 to 0.234)	0.264	-0.340 (-0.800 to 0.119)	0.145	-0.395 (-0.679 to -0.110)	0.007
Hypertension	0.240 (-0.032 to 0.511)	0.083	-0.636 (-1.168 to -0.104)	0.020	-0.452 (-0.912 to 0.007)	0.053	0.193 (-0.095 to 0.481)	0.187
CKD	0.362 (0.087 to 0.637)	0.010	-0.692 (-1.230 to -0.155)	0.012	-1.326 (-1.758 to -0.895)	< 0.0001	0.265 (-0.026 to 0.556)	0.074
Albumin	-0.194 (-0.375 to -0.013)	0.036	0.014 (-0.350 to 0.379)	0.939	-0.051 (-0.357 to 0.256)	0.743	-0.015 (-0.211 to 0.181)	0.879
Adjusted Model								
Age	0.304 (0.019 to 0.588)	0.036	-	-	-0.006 (-0.020 to 0.008)	0.428	-	-
Male- gender	-	-	0.591 (0.020 to 1.163)	0.043	-	-	-	-
BMI	-	-	-	-	-	-	-	-
Alcohol intake	-	-	-	-	-	-	-	-
Smoker	-	-	-	-	0.076 (-0.186 to 0.339)	0.565	-	-
Diabetes Mellitus	-0.320 (-0.598 to -0.041)	0.025	-	-	-0.139 (-0.578 to 0.301)	0.533	-0.492 (-0.782 to -0.202)	0.001
Hypertension	0.231 (-0.115 to 0.578)	0.189	-0.174 (-0.853 to 0.504)	0.612	0.468 (-0.058 to 0.995)	0.081	0.160 (-0.196 to 0.515)	0.375
CKD	0.256 (-0.098 to 0.609)	0.155	-0.466 (-1.131 to 0.199)	0.169	-1.509 (-2.055 to -0.963)	< 0.0001	0.270 (-0.085 to 0.625)	0.135
Albumin	-0.171 (-0.359 to 0.016)	0.072	-	-	-	-	-	-

### Altered drug metabolism in CKD patients

**Fig. 3:**
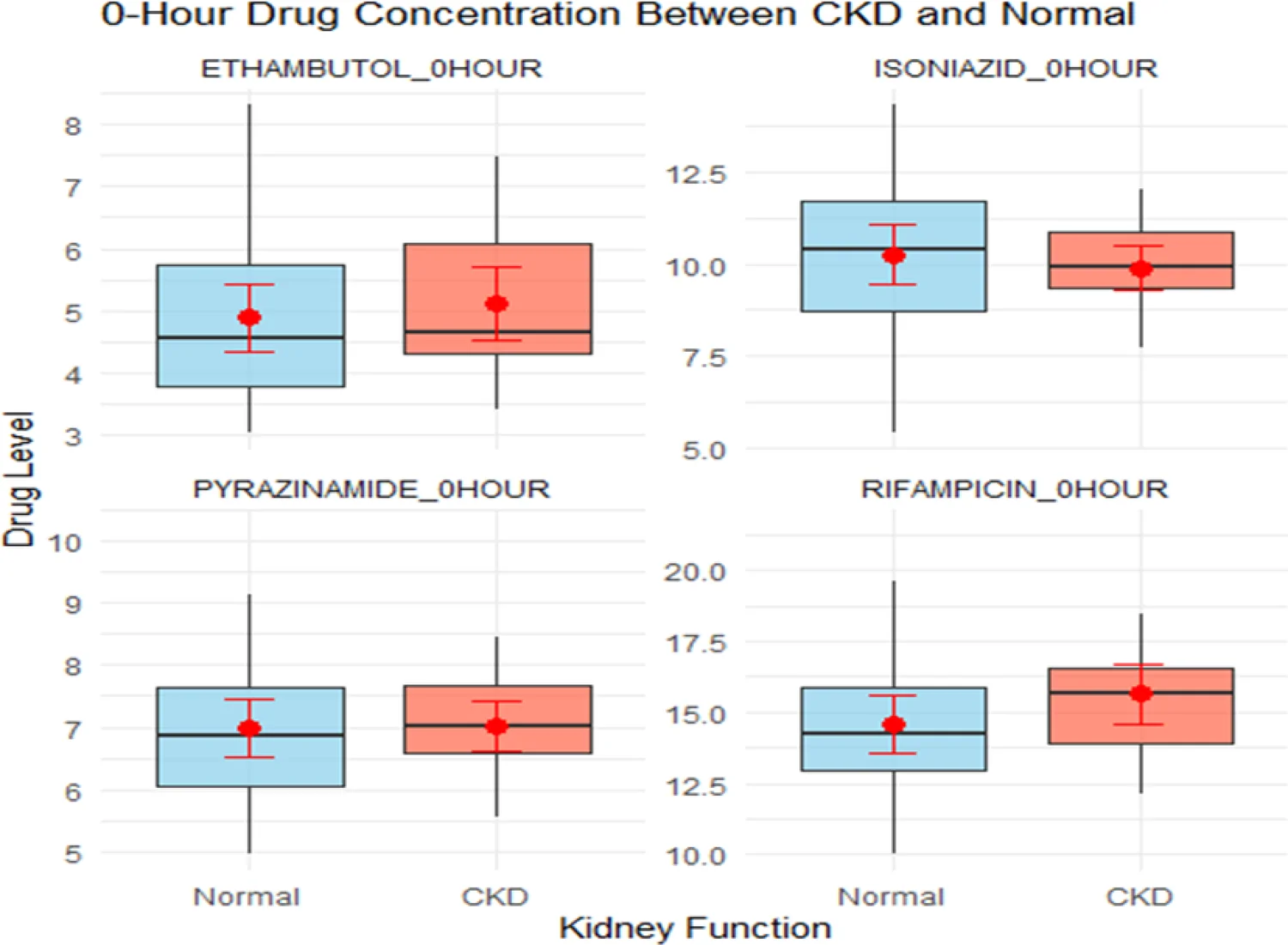
Drug concentration–time profile at trough level

**Fig. 4:**
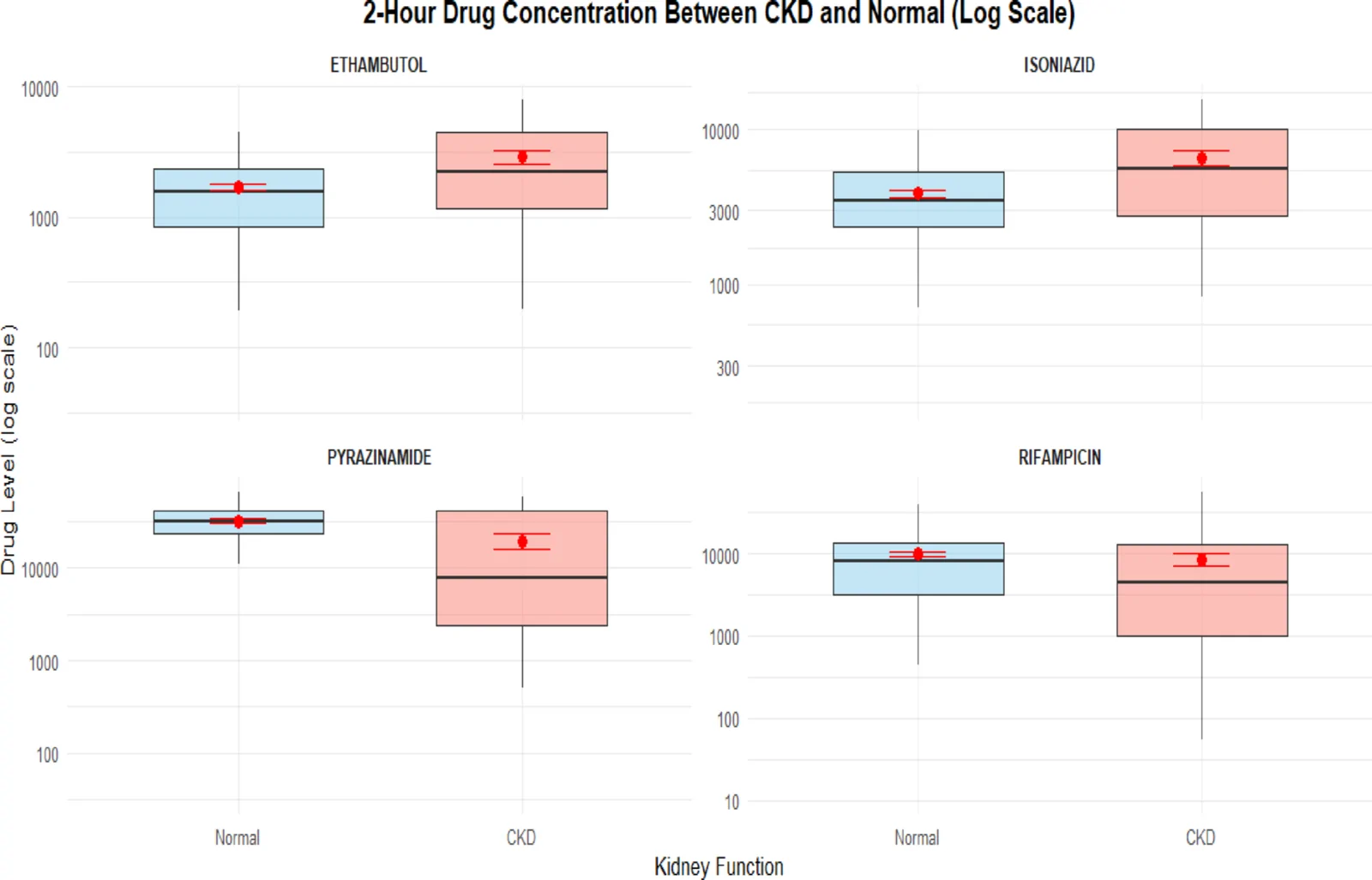
Drug concentration–time profile at 2-hour post-dose

The following outcome analysis was pre-specified as exploratory, as the study was not powered to detect independent associations between drug concentrations and clinical outcomes. Across all four first-line anti-tubercular drugs, CKD patients had higher trough concentrations of isoniazid, rifampicin, and ethambutol compared to those with normal kidney function, while pyrazinamide trough concentrations were higher in the normal kidney function group. For isoniazid, 10/51 (19.6%) of CKD patients had 2-hour concentrations exceeding 10,000 ng/mL, while concentrations in the normal kidney function group typically remained below 5,000 ng/mL; however, the difference in 2-hour concentrations did not reach statistical significance (*P* = 0.069). For rifampicin, trough concentrations were significantly higher in CKD patients (*P* < 0.0001), while 2-hour concentrations were significantly higher in the normal kidney function group (*P =* 0.033), reflecting a different cross-sectional concentration pattern between groups at the two measured time points. Pyrazinamide concentrations were significantly higher in the normal kidney function group at both time points (trough *P* = 0.002; 2-hour *P =* 0.001), with only 9/40 (22.5%) of CKD patients reaching 2-hour concentrations comparable to those observed in the normal kidney function group. Ethambutol trough and 2-hour concentrations were both significantly higher in CKD patients (*P* < 0.0001 and *P* = 0.015, respectively). These findings demonstrate that CKD is associated with measurable differences in drug concentrations at both time points; however, given the two-point sampling design, conclusions regarding absorption, clearance, accumulation, or systemic exposure cannot be drawn and observed differences in 2-hour concentrations may reflect delayed absorption rather than reduced total drug exposure, and full pharmacokinetic characterisation using multiple sampling time points is required (Figs. 3 and 4).

This plot represents plasma drug concentrations measured at trough (pre-dose) time points, indicating variability in minimum concentration levels among participants.

This figure shows plasma drug concentration levels measured 2-hours after administration across the study population.

### Adverse drug reactions and treatment outcome

The frequency of adverse drug reactions (ADRs) was comparable between the groups, with 8/51 (15.6%) in CKD and 15/100 (15%) in normal kidney function group. Drug-induced hepatitis was the most commonly reported ADR in both the groups with nearly identical frequencies of 5/51 (9.8%) in CKD and 10/100 (10%) in normal kidney function group. Optic neuritis [1/51 (1.9%)], peripheral neuropathy [1/51 (1.9%)] and psychosis [1/51 (1.9%)] were seen only in patients with CKD.

Of the 151 patients evaluated, 138 (91.3%) successfully completed their treatment and were declared cured [96/100 (96%) in the normal kidney function group vs. 42/51 (82.3%) in the CKD group, *P =* 0.011]. Mortality was significantly higher among patients with CKD compared to those with normal kidney function [12/151 (7.9%): 9/51 (17.6%) vs. 3/100 (3%), *P =* 0.003]. Only one patient was lost to follow up from normal kidney function group. Furthermore, we observed that patients with unfavourable outcomes had below established reference thresholds drug concentrations compared to those who completed their treatment but it was not significant (Supplementary table-S6).

On univariate analysis, age was significantly associated with clinical outcomes (OR 0.966, 95% CI 0.940–0.991, *P =* 0.009). However, on multivariable analysis, none of the variables, including demographic factors and anti-tubercular drug concentrations, were independently associated with clinical outcomes. For the multivariable logistic regression model of clinical outcomes, covariates were selected based on univariate association (*P* < 0.2) and included age, gender, BMI, diabetes mellitus, and the four drug concentration variables meeting this threshold (isoniazid 2-hour, rifampicin 2-hour, pyrazinamide trough, and ethambutol 2-hour). Hypertension and CKD status did not meet this threshold in univariate analysis and were therefore not included in the multivariable outcome model. (Supplementary Table-S7).

Table 6 summarizes the drug concentrations and associated covariates in patients with CKD compared to those with normal renal function.

**Table 6:** Summary of drug concentration and influencing covariates in CKD vs. normal function

Drug	Trough: CKD vs. Normal	2 h: CKD vs. Normal	Comment	Covariates affecting Trough	Covariates affecting 2 h concentration
Isoniazid	↑↑	≈≈	Significantly higher trough concentrations in CKD; no significant difference at 2-hours.	↑: Hypertension, CKD ↓: Albumin	↓: DM ↑: Age
Rifampicin	↑↑	↓↓	Higher trough concentrations but lower 2-hour concentrations in CKD compared with normal kidney function.	↓: Albumin	↑: Male
Pyrazinamide	↓↓	↓↓	Lower concentrations at both measured time points in CKD compared with normal kidney function.	↓: DM	↓: CKD
Ethambutol	↑↑	↑↑	Consistently higher concentrations observed in CKD at both measured time points.	↑ CKD ↓ DM	↓: DM

↑↑ / ↓↓ = Significantly higher/lower; ≈≈ = No significant difference; Covariates marked ↓ or ↑ depending on whether they **lower** or **increase** the drug levels; DM = Diabetes Mellitus

## Discussion

The implementation of a standard four-drug regimen under the supervision of a well-structured national TB program for the management of drug-susceptible TB has yielded cure rates exceeding 95%, while simultaneously contributing to a sustained decline in TB incidence. This therapeutic approach, having remained largely unaltered over several decades, continues to represent the globally endorsed standard of care for tuberculosis treatment [11, 16]. The current reports on TB treatment in 2021 showed 85.5% success rate in India [17]. Though there have been therapeutic targets proposed for anti-tuberculosis drugs, the existing literature on the correlation between the dose or frequency of the drug and poor outcomes from TB therapy is limited [10]. Patients with CKD require special attention, as the effectiveness of ATT depends on drug concentration. Therefore, for individuals with advanced kidney impairment (eGFR < 30 mL/min/1.73 m²) or those undergoing dialysis, extending the dosing interval rather than reducing the dose is recommended. This approach helps maintain adequate drug exposure, optimizing efficacy while minimizing the risk of drug accumulation and toxicity [8].

We found significantly higher trough concentrations of isoniazid, rifampicin, and ethambutol in CKD patients than in those with normal kidney function. Conversely, the trough concentration of pyrazinamide was higher in individuals with normal kidney function. At 2-hours post-dose, isoniazid and ethambutol concentrations remained higher in CKD patients, whereas rifampicin and pyrazinamide concentrations were significantly higher in those with normal kidney function (Table 2). This difference indicates an association between CKD and plasma drug concentrations. However, owing to the limited two-point sampling design, the relative contributions of absorption, distribution, metabolism, and elimination cannot be determined. After adjusting for 2-hour concentrations, CKD status remained independently associated with trough concentrations of isoniazid, rifampicin, and ethambutol, but not pyrazinamide. These findings are presented as statistical associations; as trough concentrations are measured prior to dosing, no causal inference between 2-hour and trough concentrations is implied. In addition, 2-hour concentrations were significantly associated with trough levels across all four drugs, highlighting the relationship between early drug exposure and sustained pre-dose concentrations (Table 3). An important limitation of between-group comparisons in this study is the baseline imbalance between CKD and normal kidney function groups: CKD patients were older and had significantly higher prevalences of hypertension and diabetes mellitus, all of which independently influence drug concentration. Although key confounders were included in the multivariable regression models, residual confounding cannot be excluded, and the observed differences in drug concentrations between groups should be interpreted carefully.

As rifampicin is bound to plasma proteins at a rate of 60–80%, patients with reduced albumin levels may demonstrate lower drug concentrations that could be attributable to altered plasma protein binding. Lower albumin levels allow a larger fraction of the drug to be distributed and cleared from the plasma, possibly resulting in insufficient exposure to anti-bacterial agents and challenges in reaching pharmacodynamic goals. In this study, higher serum albumin was independently associated with significantly lower trough concentrations of isoniazid and rifampicin, which may reflect increased protein binding of these drugs in patients with higher albumin levels, reducing the free drug fraction available for distribution into tissues and thereby lowering measurable plasma concentrations. CKD was found to be a strong independent predictor of altered anti-TB drug levels being associated with elevated concentrations of isoniazid and ethambutol at baseline, and 2-hour reduced levels of pyrazinamide highlighting the complex effects of kidney impairment on drug concentration, even for drugs primarily metabolized by the liver. Additionally, the presence of diabetes mellitus was significantly associated with reduced trough concentrations of pyrazinamide and ethambutol, and with reduced 2-hour concentrations of ethambutol. These findings highlight the importance of considering underlying comorbidities, particularly CKD, albumin level, and diabetes, when interpreting drug levels and optimizing dosing in patients receiving first-line anti-tubercular therapy. The lower trough and 2-hour pyrazinamide concentrations observed in CKD patients compared to those with normal kidney function warrants further consideration, as pyrazinamide and its metabolites are primarily renally cleared, making accumulation in CKD theoretically more likely. Several mechanisms may account for this observation. CKD-associated alterations in gastrointestinal motility and intestinal drug transporter expression may impair oral absorption of pyrazinamide [18, 19]. Additionally, fluid retention and altered body composition in advanced CKD may increase the apparent volume of distribution of pyrazinamide, which could result in lower measured plasma concentrations [20, 21]. Furthermore, in patients with eGFR < 30 mL/min/1.73 m² receiving alternate-day pyrazinamide and ethambutol dosing, the trough sample reflects a longer 48-hour post-dose interval compared with the 24-hour interval in daily-dosed patients, which may itself contribute to the lower pyrazinamide and ethambutol concentrations observed in this subgroup, independent of any true CKD-related pharmacokinetic change; sampling timing alone, however, does not fully explain the observed differences. These remain plausible explanations rather than confirmed mechanisms, given the two-point sampling design. The marked elevation in isoniazid trough concentrations observed in CKD patients raises the possibility of an interplay between impaired renal function and non-renal metabolic clearance pathways. Although isoniazid is primarily metabolised hepatically via NAT2-dependent acetylation, uraemic retention solutes in advanced CKD may potentially inhibit hepatic enzyme activity, which may contribute to higher measured isoniazid concentrations beyond what would be expected from reduced renal clearance alone [22–24]. However, acetylator status was not assessed in this study; therefore, the relative contribution of NAT2 phenotype versus CKD-related metabolic alterations cannot be determined and is acknowledged as a limitation. Future studies incorporating NAT2 genotyping are needed to better characterise isoniazid pharmacokinetics in CKD populations.

Also, we observed that advanced stages of CKD, particularly stages 4 and 5, significantly altered the drug concentration of first-line anti-tuberculosis drugs. The changes included elevated baseline concentrations that is trough of isoniazid, rifampicin, pyrazinamide and ethambutol in stage 5, as well as reduced 2-hour levels of rifampicin and pyrazinamide, suggesting differences in measured concentrations between CKD stages and normal kidney function. As above, the limited sampling strategy precludes distinguishing between these underlying processes. These stage-specific differences reinforce the potential value of individualized monitoring in advanced CKD. Furthermore, the small number of patients in CKD stages 2 and 3 limits the precision of stage-specific estimates, and these subgroup findings should be interpreted with caution. (Supplementary Table-S2 & S3)

We also examined the prevalence of below the reference threshold in patients with CKD and normal kidney function. We found that 2-hour post-dose concentrations of rifampicin and pyrazinamide were below the reference threshold in CKD patients compared to those with normal kidney function (Fig. 2). It is important to note that these reference thresholds were derived predominantly from studies in normal kidney function populations, and their validity as markers of therapeutic adequacy in CKD patients remains uncertain. Although concentrations below reference thresholds were prevalent in a substantial proportion of patients, the majority completed treatment and were declared cured at the end of treatment. This observation raises an important question as to whether the established reference thresholds and dosing recommendations require revision specifically for patients with CKD. Further studies in patients with CKD and other comorbidities are required to confirm these findings and validate appropriate therapeutic targets for this population.

The observation that a substantial proportion of patients had 2-hour drug concentrations below established reference thresholds, yet the majority successfully completed treatment and were declared cured, warrants critical consideration. It is possible that these thresholds are overly conservative in CKD, or alternatively that 2-hour concentration alone is an insufficient predictor of microbiological cure, which is determined by multiple additional factors including the minimum inhibitory concentration of the infecting organism, duration and pattern of drug exposure over the full AUC, and host immune function. These findings reinforce the need for prospective validation of CKD-specific pharmacokinetic and pharmacodynamic targets before firm conclusions about therapeutic adequacy can be drawn in this population.

Other studies on therapeutic drug monitoring in patients with normal kidney function have reported plasma concentrations below the recommended threshold, with rifampicin showing a prevalence of 60–66% and isoniazid 54–55%. Other studies indicated even higher rates, ranging from 71 to 100% for rifampicin and 29–84% for isoniazid. Concentrations below reference thresholds were less common for pyrazinamide (3–13%) and ethambutol (31–46%) [10, 11, 25–27]. In contrast to these findings, our study demonstrated below the reference threshold concentrations in 31.8% of patients for pyrazinamide and 67.0% for ethambutol in individuals with normal kidney function. Despite these lower drug levels, the majority of patients in our study 138 (91.3%) completed treatment and were declared cured by the treating physician, and were therefore considered cured, which is notably higher than the 68.6% cure rate reported in another study [11].

In our study, while the overall incidence of ADRs was similar between patients with normal kidney function and those with CKD, distinct differences were observed in the type and severity of ADRs. Drug-induced hepatitis was noted in both groups, reflecting the hepatotoxic potential of anti-tubercular therapy; however, patients with CKD exhibited a unique vulnerability to neurotoxic effects, such as optic neuritis and psychosis, which were absent in patients with normal kidney function. This may reflect elevated plasma concentrations of certain drugs or their metabolites related to impaired kidney clearance in CKD, increasing the risk of specific toxicities. Although the frequency of ADRs was not significantly higher in CKD, the presence of more serious and potentially irreversible reactions underscores the need for careful therapeutic monitoring and individualized dosing. These findings highlight the importance of considering kidney function, comorbidities, and drug pharmacokinetics when managing anti-tubercular treatment to minimize the risk of ADRs.

The relationship between drug concentrations and clinical outcomes was examined as an exploratory analysis, as the study was not powered for outcome prediction. Mortality was significantly higher in CKD patients compared to those with normal kidney function [9/51 (17.6%) vs. 3/100 (3.0%), *P =* 0.003]. Patients with unfavourable outcomes had numerically lower drug concentrations compared to those who completed treatment, though this difference did not reach statistical significance. On multivariable analysis, none of the variables including demographic factors and drug concentrations were independently associated with clinical outcomes. These findings should therefore be regarded as hypothesis-generating, and future adequately powered studies are required to determine whether drug concentrations independently predict outcomes in TB patients with CKD.

To our knowledge, this is the first systematic study assessing the drug concentration of first-line TB therapy drugs in CKD patients (Stage 2–5) compared to patients with normal renal function. Of the 488 patients screened, 151 were ultimately included in the study (Fig. 1). The remaining 337 were excluded at the time of screening: 122 were not willing to participate, 48 were lost to follow-up (did not return after screening or were unavailable for enrolment), 43 wished to follow up at a local NTEP centre, 38 were HIV-positive, 37 were aged < 18 or > 70 years, 27 had MDR-TB or were on modified ATT, 11 had ATT modified before two weeks, 7 died before enrolment, and 4 were non-adherent to medication. As baseline characteristics of patients who declined participation or were lost before enrolment were not systematically collected, it was not possible to determine whether they differed from those included in the study. Consequently, the possibility of selection bias cannot be excluded, and the findings may not be fully generalisable to the broader population of patients with tuberculosis, particularly those with CKD. The shortcomings of this study are its small number of subjects as well as insufficient statistical power for identifying significant differences in the percentage of subjects with adverse reactions among the two groups. The two-point sampling strategy employed precludes calculation of full pharmacokinetic parameters such as AUC, Cmax, and half-life, and the 2-hour post-dose concentration may not accurately reflect maximum drug exposure in patients with delayed gastric emptying, which is a known complication of both advanced CKD and diabetes mellitus. Concentrations below the established 2-hour reference threshold in these patients may therefore reflect delayed absorption rather than genuinely reduced total drug exposure, and AUC-based assessments using multiple sampling time points would be required to distinguish between these possibilities. Patients were sampled over a relatively broad treatment interval (median 30 days; IQR 18–47 days); although all had completed a minimum of two weeks of treatment, variability in exact treatment duration at sampling could still influence measured concentrations, and this variable was not incorporated as a covariate in the regression analyses. Acetylator status was not assessed, limiting interpretation of isoniazid accumulation. It is also important to acknowledge that HIV-positive patients were excluded from this study to avoid pharmacokinetic confounding from antiretroviral therapy, particularly the well-established interaction between rifampicin and antiretroviral agents. While this decision improves the internal validity of the pharmacokinetic comparisons, it limits the generalisability of our findings to settings with a high TB/HIV co-infection burden, where CKD is also prevalent. Future studies should specifically examine the pharmacokinetics of first-line ATT in patients with concurrent CKD and HIV infection. Despite these limitations, our findings support the value of individualized drug concentration monitoring in patients with TB and comorbid conditions.

## Conclusion

CKD significantly alters the plasma concentrations of all four first-line anti-tubercular drugs, with distinct and drug-specific patterns. Trough concentrations of isoniazid, rifampicin, and ethambutol were significantly higher in CKD patients compared with those with normal kidney function, indicating altered drug concentration profiles in this population. In contrast, 2-hour post-dose concentrations of rifampicin and pyrazinamide were significantly lower in CKD patients, with a higher proportion falling below established reference thresholds. CKD was identified as an independent predictor of altered drug concentrations for multiple first-line agents. Higher serum albumin was independently associated with lower trough concentrations of isoniazid and rifampicin, while diabetes mellitus was associated with reduced concentrations of pyrazinamide and ethambutol. Mortality was significantly higher in CKD patients. These findings collectively highlight the importance of therapeutic drug monitoring and individualised dosing strategies in TB patients with CKD, particularly those with advanced renal impairment, albumin levels, and diabetes mellitus. Future multi-timepoint drug level studies may bring further clarity.

## Supplementary Information

Below is the link to the electronic supplementary material.

Supplementary Material 1

## Data Availability

No datasets were generated or analysed during the current study.

## References

[1] Evaluation CKD. and Management – KDIGO [Internet]. [cited 2024 Oct 2]. Available from: https://kdigo.org/guidelines/ckd-evaluation-and-management/

[2] India TBR. 2023 – Central Tuberculosis Division [Internet]. [cited 2024 Sep 17]. Available from: https://tbcindia.mohfw.gov.in/2023/06/06/india-tb-report-2023/

[3] Saito N, Yoshii Y, Kaneko Y, Nakashima A, Horikiri T, Saito Z, et al. Impact of renal function-based anti-tuberculosis drug dosage adjustment on efficacy and safety outcomes in pulmonary tuberculosis complicated with chronic kidney disease. BMC Infect Dis. 2019;19(1):374. 10.1186/s12879-019-4010-7 . PubMed PMID: 31046706; PubMed Central PMCID: PMC6498605. PMC6498605 31046706

[4] National Institute for Health and Care Excellence. The Guidelines Manual [Internet]. London: National Institute for Health and Care Excellence (NICE); 2012 [cited 2023 Aug 30]. (NICE Process and Methods Guides). Available from: http://www.ncbi.nlm.nih.gov/books/NBK395866/ PubMed PMID: 27905714. 27905714

[5] Cho PJY, Wu CY, Johnston J, Wu MY, Shu CC, Lin HH. Progression of chronic kidney disease and the risk of tuberculosis: an observational cohort study. Int J Tuberc Lung Dis. 2019;23(5):555–62. 10.5588/ijtld.18.0225 . PubMed PMID: 31097063. 31097063

[6] Shen TC, Huang KY, Chao CH, Wang YC, Muo CH, Wei CC, et al. The risk of chronic kidney disease in tuberculosis: a population-based cohort study. QJM. 2015;108(5):397–403. 10.1093/qjmed/hcu220 . PubMed PMID: 25352683. 25352683

[7] Gurumurthy P, Raghupati Sarma G, Jayasankar K, Thyagarajan K, Prabhakar R, Muthusethupathi MA, et al. Single-dose pharmacokinetics of isoniazid and rifampicin in patients with chronic renal failure. Indian J Tuberculosis. 1992;39(4):221–8.

[8] Peloquin CA. Therapeutic Drug Monitoring in the Treatment of Tuberculosis. Drugs. 2002;62(15):2169–83. 10.2165/00003495-200262150-00001. 12381217

[9] Munar MY, Singh H. Drug dosing adjustments in patients with chronic kidney disease. Am Fam Physician. 2007;75(10):1487–96. PubMed PMID: 17555141. 17555141

[10] Ramachandran G, Chandrasekaran P, Gaikwad S, Agibothu Kupparam HK, Thiruvengadam K, Gupte N, et al. Subtherapeutic Rifampicin Concentration Is Associated With Unfavorable Tuberculosis Treatment Outcomes. Clin Infect Dis. 2020;70(7):1463–70. 10.1093/cid/ciz380 . PubMed PMID: 31075166; PubMed Central PMCID: PMC7931830. PMC7931830 31075166

[11] Trentalange A, Borgogno E, Motta I, Antonucci M, Pirriatore V, Costa C, et al. Rifampicin and Isoniazid Maximal Concentrations are Below Efficacy-associated Thresholds in the Majority of Patients: Time to Increase the Doses? Int J Antimicrob Agents. 2021;57(3):106297. 10.1016/j.ijantimicag.2021.106297. 33539932

[12] Datta D, Rao IR, Prabhu AR, Nagaraju SP, Thunga G, Magazine R, et al. Effect of chronic kidney disease on adverse drug reactions to anti-tubercular treatment: a retrospective cohort study. Ren Fail. 2024;46(2):2392883. 2024.2392883 PubMed PMID: 39165235; PubMed Central PMCID: PMC11340219. 10.1080/0886022X.2024.2392883 PMC11340219 39165235

[13] Guideline for PMDT in India. 2017 – Central Tuberculosis Division [Internet]. [cited 2024 Sep 24]. Available from: https://tbcindia-wp.azurewebsites.net/guideline-for-pmdt-in-india-2017-3/

[14] Kidney Disease: Improving Global Outcomes (KDIGO) CKD Work Group. KDIGO 2012 Clinical Practice Guideline for the Evaluation and Management of Chronic Kidney Disease. Kidney Int Suppl. 2013;3(1):1–150. Available from: https://kdigo.org/wp-content/uploads/2017/02/KDIGO_2012_CKD_GL.pdf

[15] Alsultan A, Peloquin CA. Therapeutic drug monitoring in the treatment of tuberculosis: an update. Drugs. 2014;74(8):839–54. 10.1007/s40265-014-. 0222-8 PubMed PMID: 24846578. 24846578

[16] Infectious Diseases Society of America. Treatment of tuberculosis. MMWR Recomm Rep. 2003;52(RR–11):1–77. 12836625

[17] Central Tuberculosis Division. India TB Annual Report 2023. New Delhi: Ministry of Health and Family Welfare, Government of India. 2023. Available from: https://tbcindia.mohfw.gov.in

[18] Vaziri ND, Yuan J, Rahimi A, Ni Z, Said H, Subramanian VS. Disintegration of colonic epithelial tight junction in uremia: a likely cause of CKD-associated inflammation. Nephrol Dial Transpl. 2012;27(7):2686–93. 10.1093/ndt/gfr624 . PubMed PMID: 22131233; PubMed Central PMCID: PMC3616758. PMC3616758 22131233

[19] Murtagh FEM, Addington-Hall J, Higginson IJ. The Prevalence of Symptoms in End-Stage Renal Disease: A Systematic Review. Adv Chronic Kidney Dis. 2007;14(1):82–99. 10.1053/j.ackd.2006.10.001 . PubMed PMID: 17200048. 17200048

[20] Dreisbach AW, Lertora JJL. The effect of chronic renal failure on drug metabolism and transport. Expert Opin Drug Metab Toxicol. 2008;4(8):1065–74. 10.1517/17425255.4.8.1065 . PubMed PMID: 18680441; PubMed Central PMCID: PMC2745294. PMC2745294 18680441

[21] Nolin TD, Unruh ML. Clinical relevance of impaired nonrenal drug clearance in ESRD. Semin Dial. 2010;23(5):482–5. 10.1111/j.1525-139x.2010. 00775.x PubMed PMID: 21069923. 21069923

[22] Donald PR, Parkin DP, Seifart HI, Schaaf HS, van Helden PD, Werely CJ, et al. The influence of dose and N-acetyltransferase-2 (NAT2) genotype and phenotype on the pharmacokinetics and pharmacodynamics of isoniazid. Eur J Clin Pharmacol. 2007;63(7):633–9. 10.1007/s00228-007-0305-5 . PubMed PMID: 17505821. 17505821

[23] Azuma J, Ohno M, Kubota R, Yokota S, Nagai T, Tsuyuguchi K, et al. NAT2 genotype guided regimen reduces isoniazid-induced liver injury and early treatment failure in the 6-month four-drug standard treatment of tuberculosis: A randomized controlled trial for pharmacogenetics-based therapy. Eur J Clin Pharmacol. 2013;69(5):1091–101. 10.1007/s00228-012-1429-9. PMC3641305 23150149

[24] Nolin TD, Naud J, Leblond FA, Pichette V. Emerging evidence of the impact of kidney disease on drug metabolism and transport. Clin Pharmacol Ther. 2008;83(6):898–903. 10.1038/clpt.2008.59 . PubMed PMID: 18388866. 18388866

[25] Shaheen A, Najmi MH, Saeed W, Farooqi ZUR. Pharmacokinetics of standard dose regimens of rifampicin in patients with pulmonary tuberculosis in Pakistan. Scand J Infect Dis. 2012;44(6):459–64. 10.3109/00365548.2011.647832 . PubMed PMID: 22224832. 22224832

[26] Prahl JB, Johansen IS, Cohen AS, Frimodt-Møller N, Andersen ÅB. Clinical significance of 2 h plasma concentrations of first-line anti-tuberculosis drugs: a prospective observational study. J Antimicrob Chemother. 2014;69(10):2841–7. 10.1093/jac/dku210 . PubMed PMID: 25140577. 25140577

[27] Sekaggya-Wiltshire C, von Braun A, Scherrer AU, Manabe YC, Buzibye A, Muller D, et al. Anti-TB drug concentrations and drug-associated toxicities among TB/HIV-coinfected patients. J Antimicrob Chemother. 2017;72(4):1172–7. 10.1093/jac/dkw534 . PubMed PMID: 28108678; PubMed Central PMCID: PMC5703350. PMC5703350 28108678

