# Supplementary material for: Effect of kidney function on drug concentrations of first-line anti-tubercular treatment
Source: Infection. 2026 Aug 31;54(5):2835–50. doi: 10.1007/s15010-026-02931-7 (PMC13727858; doi:10.1007/s15010-026-02931-7)
Supplement: Supplementary file 1 [file 15010_2026_2931_MOESM1_ESM.docx]

**Supplementary Table: S1-** **Comparison of first-line anti-tubercular drug concentration in CKD and normal kidney function Difference delta (2-hour minus trough)**

| **Drug, Median (IQR), ng/mL** | **CKD (n=51)** | **Normal kidney function (n=100)** | **P value** |
| --- | --- | --- | --- |
| **Isoniazid** | 3583.48 (1218.49–5491.08) | 3122.82 (1752.28–4949.88) | 0.998 |
| **Rifampicin** | 2962.27 (377.64–12588.78) | 7789.65 (3109.42–13365.87) | **0.009** |
| **Pyrazinamide** | 5629.09 (1658.36–36917.68) | 26112.87 (15426.52–35965.12) | **0.002** |
| **Ethambutol** | 1172.05 (381.23–2678.77) | 1146.52 (548.40–1964.05) | 0.832 |

**Supplementary Table-S2: - Comparison drug concentration between different stages of CKD**

| **Drugs, Median (IQR), ng/mL** | **Normal kidney Function (n=100)** | **Stage 2**  **(n=6)** | **Stage 3 (n=7)** | **Stage 4 (n=13)** | **Stage 5 (n=25)** | **P-value** |
| --- | --- | --- | --- | --- | --- | --- |
| **Isoniazid Trough (ng/mL)** | 187.58 (95.56–323.65) | 476(269–581) | 705 (491–762) | 1050 (357–4226) | 2306 (675–4376) | **0.001** |
| **Isoniazid 2-Hour (ng/mL)** | 3435.60 (2280.25–5456.88) | 5376 (4709–6553) | 3217 (3108–3703) | 5632(3679–10883) | 5381 (2011–10105) | 0.123 |
| **Rifampicin Trough (ng/mL)** | 33.24 (22.49–86.20) | 117 (36.0–200) | 64.4 (44.2–124) | 140 (53.5–755) | 302 (89.9–795) | **<0.0001** |
| **Rifampicin 2-Hour (ng/mL)** | 8114.65 (3178.24–13602.36) | 16533 (9702–25138) | 3350 (789–62420) | 5480 (2330–13891) | 3334 (988–7661) | **0.021** |
| **Pyrazinamide Trough (ng/mL)** | 4273.94 (2151.29–8023.81) | 5802 (5045–11012) | 5417 (3270–7563) | 3143 (2510–3414) | 5118 (2620–7233) | **<0.0001** |
| **Pyrazinamide 2-Hour (ng/mL)** | 32216.62 (23267.01–42096.23) | 43632 (15410–53241) | 6050 (4720–16394) | 3196 (1633–26587) | 7488 (1745–41569) | **<0.0001** |
| **Ethambutol Trough (ng/mL)** | 230.92 (161.95–417.22) | 391 (157–515) | 804 (624–1087) | 738 (296–1427) | 1577 (400–2343) | **0.001** |
| **Ethambutol 2-Hour (ng/mL)** | 1589.37 (838.42–2441.28) | 2223 (596–3727) | 1735 (1177–2128) | 1158 (645–3622) | 3012 (1537–5234) | **0.010** |

**Supplementary Table-S3: - post-hoc analysis for comparison of drug concentration within the stages**

| **Drugs** | **Stages** | **P- value** |
| --- | --- | --- |
| **Isoniazid Trough (ng/mL)** | NKF-Stage 2 | 0.298 |
|  | NKF- Stage 3 | 0.418 |
|  | NKF- Stage 4 | **0.019** |
|  | NKF-Stage 5 | **0.024** |
|  | | |
| **Isoniazid 2-Hour (ng/mL)** | NKF-Stage 2 | 0.439 |
|  | NKF- Stage 3 | 0.907 |
|  | NKF- Stage 4 | 0.829 |
|  | NKF-Stage 5 | 0.305 |
|  | | |
| **Rifampicin Trough (ng/mL)** | NKF-Stage 2 | 0.849 |
|  | NKF- Stage 3 | 0.334 |
|  | NKF- Stage 4 | 0.492 |
|  | NKF-Stage 5 | **<0.0001** |
|  | | |
| **Rifampicin 2-Hour (ng/mL)** | NKF-Stage 2 | 0.561 |
|  | NKF- Stage 3 | 0.579 |
|  | NKF- Stage 4 | 0.992 |
|  | NKF-Stage 5 | **0.039** |
|  | | |
| **Pyrazinamide Trough (ng/mL)** | NKF-Stage 2 | 0.655 |
|  | NKF- Stage 3 | 0.210 |
|  | NKF- Stage 4 | **0.002** |
|  | NKF-Stage 5 | **0.002** |
|  | | |
| **Pyrazinamide 2-Hour (ng/mL)** | NKF-Stage 2 | 0.855 |
|  | NKF- Stage 3 | 0.140 |
|  | NKF- Stage 4 | **0.031** |
|  | NKF-Stage 5 | **0.001** |
|  | | |
| **Ethambutol Trough (ng/mL)** | NKF-Stage 2 | 0.987 |
|  | NKF- Stage 3 | **0.009** |
|  | NKF- Stage 4 | 0.148 |
|  | NKF-Stage 5 | **0.021** |
|  | | |
| **Ethambutol 2-Hour (ng/mL)** | NKF-Stage 2 | 0.983 |
|  | NKF- Stage 3 | 0.999 |
|  | NKF- Stage 4 | 1.000 |
|  | NKF-Stage 5 | **0.002** |

**Supplementary Table S4: Sensitivity analysis of eGFR (continuous) and trough drug concentrations**

| **Drug** | **Unadjusted β (95% CI)** | **P value** | **Adjusted β (95% CI)** | **P value** |
| --- | --- | --- | --- | --- |
| **Isoniazid** | −0.018 (−0.022 to −0.013) | **<0.001** | −0.012 (−0.019 to −0.005) | **0.001** |
| **Rifampicin** | −0.013 (−0.018 to −0.008) | **<0.001** | −0.009 (−0.017 to −0.002) | **0.014** |
| **Pyrazinamide** | 0.001 (−0.004 to 0.007) | 0.674 | −0.0001 (−0.005 to 0.005) | 0.997 |
| **Ethambutol** | −0.012 (−0.015 to −0.009) | **<0.001** | −0.009 (−0.013 to −0.004) | **<0.001** |

**Supplementary Table S5: Sensitivity analysis of eGFR (continuous) and 2-hour post-dose drug concentrations**

| **Drug** | **Unadjusted β (95% CI)** | **P value** | **Adjusted β (95% CI)** | **P value** |
| --- | --- | --- | --- | --- |
| **Isoniazid** | −0.003 (−0.005 to 0.000) | **0.074** | 0.001 (−0.003 to 0.005) | 0.711 |
| **Rifampicin** | 0.007 (0.002 to 0.013) | **0.009** | 0.006 (−0.001 to 0.012) | 0.087 |
| **Pyrazinamide** | 0.015 (0.011 to 0.020) | **<0.0001** | 0.018 (0.012 to 0.024) | **<0.0001** |
| **Ethambutol** | −0.003 (−0.006 to −0.001) | **0.021** | −0.004 (−0.007 to 0.000) | **0.037** |

**Supplementary Table-S6: Drug concentration based on treatment outcome**

| **Drug, Median (IQR), ng/mL** | **Favourable outcome (n=138)** | **Unfavourable outcome (n=13)** | **P value** |
| --- | --- | --- | --- |
| **Isoniazid Trough** | 250.9 (103.0– 675.4) | 205.8 (95.7– 668.7) | 0.853 |
| **Isoniazid 2-Hour** | 3729.5 (2383.4– 6028.0) | 3198.8 (1555.2– 5575.9) | 0.195 |
| **Rifampicin Trough** | 43.8 (24.2– 189.6) | 32.7 (21.8– 170.1) | 0.331 |
| **Rifampicin 2-Hour** | 7635.4 (2820.6– 13561.5) | 5337.6 (1883.2– 11149.7) | 0.163 |
| **Pyrazinamide Trough** | 4471.0 (1741.0– 8055.5) | 3620.6 (2044.3– 5952.7) | 0.943 |
| **Pyrazinamide 2-Hour** | 27354.8 (3397.8– 41412.7) | 24871.2 (7685.5– 31578.0) | 0.586 |
| **Ethambutol Trough** | 323.2 (184.0– 651.9) | 236.0 (154.8–560.9) | 0.310 |
| **Ethambutol 2-Hour** | 1693.5 (1088.8–2779.3) | 1502.4 (726.8, 2763.6) | 0.238 |

**Supplementary Table-S7: Univariate and multivariable logistic regression analysis of factors associated with clinical outcomes**

| **Univariate analysis** | | | **Multivariate analysis** | |
| --- | --- | --- | --- | --- |
| **Covariates** | **β(95%CI)** | **P value** | **β(95%CI)** | **P value** |
| **Age** | 0.966 (0.940-0.991) | **0.009** | 0.978 (0.946 – 1.011) | 0.184 |
| **Female- gender** | 2.055 (0.861- 4.905) | **0.105** | 1.295 (0.467 – 3.591) | 0.620 |
| **BMI** | 1.091 (0.991-1.201) | **0.077** | 1.114 (0.996 – 1.247) | 0.060 |
| **Alcohol Intake** | 1.258 (0.495 – 3.197) | 0.629 | - | - |
| **Smoking** | 0.693 (0.284 – 1.688) | 0.419 | - | - |
| **Diabetes Mellitus** | 0.499 (0.235 – 1.057) | **0.069** | 0.518 (0.194 – 1.384) | 0.189 |
| **Hypertension** | 0.939 (0.438 – 2.016) | 0.872 | - | - |
| **CKD** | 1.345 (0.604 – 2.994) | 0.468 | - | - |
| **Isoniazid Trough** | 1.561 (0.498 – 4.894) | 0.445 | - | - |
| **Isoniazid 2-Hour** | 0.578 (0.272 – 1.225) | **0.152** | 0.609 (0.232 – 1.598) | 0.314 |
| **Rifampicin Trough** | 3.027 (0.185 – 49.61) | 0.438 | - | - |
| **Rifampicin 2-Hour** | 0.594 (0.279 – 1.264) | **0.176** | 0.584 (0.224- 1.524) | 0.272 |
| **Pyrazinamide Trough** | 3.143 (0.607 – 16.28) | **0.172** | 5.639 (0.791 – 40.06) | 0.084 |
| **Pyrazinamide 2-Hour** | 1.172 (0.558 – 2.463) | 0.675 | - | - |
| **Ethambutol Trough** | 1.782 (0.491- 6.457) | 0.379 | - | - |
| **Ethambutol 2-Hour** | 0.532 (0.241 – 1.177) | **0.119** | 0.660 (0.234 – 1.865) | 0.433 |
